# 14-3-3β Promotes Migration and Invasion of Human Hepatocellular Carcinoma Cells by Modulating Expression of MMP2 and MMP9 through PI3K/Akt/NF-κB Pathway

**DOI:** 10.1371/journal.pone.0146070

**Published:** 2016-01-05

**Authors:** Yufu Tang, Pengfei Lv, Zhongyi Sun, Lei Han, Wenping Zhou

**Affiliations:** 1 Department of Hepatobiliary Surgery, The General Hospital of Shenyang Military Area Command, Shenyang, People’s Republic of China; 2 Post-doctoral Station, The General Hospital of Shenyang Military Area Command, Shenyang, People’s Republic of China; University of Quebec at Trois-Rivieres, CANADA

## Abstract

14-3-3β has been demonstrated to possess the oncogenic potential, and its increased expression has been detected in multiple types of carcinomas. However, majority of previous studies focused on the role of 14-3-3β in tumor cell proliferation and apoptosis, leaving much to be elucidated about its function in tumor cell invasion and metastasis. Hence, the present study aimed to investigate the role of 14-3-3β in the invasion of hepatocellular carcinoma (HCC) cells and the implications in the prognosis of HCC patients. We first examined the expression of 14-3-3β in the primary tumors of HCC patients with or without portal vein tumor thrombus (PVTT), and found that 14-3-3β expression was higher in the primary tumors with PVTT, and the level was even higher in the PVTTs. Kaplan-Meier curves and multivariate analysis revealed that high expression of 14-3-3β was associated with overall survival (OS) and time to recurrence (TTR) of HCC patients. In addition, ectopic expression of 14-3-3β in HCC cell lines led to enhanced migration ability and invasiveness, as well as up-regulation of matrix metalloproteinase 2 and 9, which could be suppressed by inhibiting the activation of Akt and nuclear factor-κB (NF-κB) signaling. Furthermore, we identified a correlated elevation of 14-3-3β and p-Akt in the primary tumors of HCC patients, and showed that a combinatory detection of 14-3-3β and p-Akt could better predict post-surgical outcome of HCC patients.

## Introduction

Hepatocellular carcinoma (HCC) is one of most common cancers worldwide [1]. Despite the advanced modalities that are commonly applied in HCC patients, such as hepatic resection, liver transplantation, transcatheter arterial chemoembolization (TACE) and ablation therapy, the prognosis remains extremely poor [2]. The 5-year survival rate is less than 30% in HCC patients after surgical resection, mainly because of the high recurrence and metastasis rates. However, the mechanisms underlying the recurrence and metastasis in HCC still remain unclear. Hence, further understanding of the underlying mechanisms is crucial for the development of novel therapeutic strategies, and would thereby improve the prognosis of HCC patients.

14-3-3 proteins are a well-known family of highly conserved proteins that contain seven distinct isoforms (β, γ, ε, ζ, η, σ, and τ) in mammals [3–5]. These proteins lack endogenous enzymatic activity and exert their functions by directly binding to their target proteins. Generally, 14-3-3 target proteins, which contain phospho-serine/threonine motifs, are regulated by 14-3-3 through alterations in protein conformation, stability, catalytic activity, subcellular localization, and complex formation. Not surprisingly, 14-3-3 proteins play central roles in regulating various biological pathways, such as those controlling cell cycle, protein trafficking, apoptosis, metabolism, signal transduction, inflammation and cell adhesion/motility [6]. Furthermore, recent studies have revealed that 14-3-3 proteins are involved in the pathogenesis of a broad range of diseases, particularly in multiple types of cancer [7,8]. Among the 14-3-3 proteins, 14-3-3σ is considered to be a tumor suppressor, andother 14-3-3 isoforms are thought to play oncogenic roles in multiple tumors [8,9].

Accumulating evidences suggest that 14-3-3β plays an important role in tumorigenesis and tumor progression. For example, increased expression of 14-3-3β has been observed in a large number of solider tumors, including lung cancer [10], astrocytoma [11], glioma [12], squamous cell carcinoma [13], colorectal cancer [14], gastric cancer [15] and HCC [16]. Overexpression of 14-3-3β in NIH 3T3 cells has been identified to stimulate anchorage-independent growth and tumor formation in nude mice [17]. Reduction of 14-3-3β expression in rat hepatoma AFB1-K2 cells by forced expression of antisense 14-3-3β RNA significantly suppressed tumor cell proliferation and tumorigenesis [18], suggesting a pivotal role of 14-3-3β in the abnormal growth of tumor cells. Recently, Liu *et al*. reported that elevated 14-3-3β in HCC was a risk factor for extrahepatic metastasis and predicted a worse 5-year survival rate [16]. Nevertheless, the molecular mechanism underlying the role of 14-3-3β in HCC metastasis and progression remains elusive.

In the currently study, we found that high 14-3-3β protein expression in primary HCC tissues was associated with significantly worse clinical outcomes. The *in vitro* Transwell assays revealed that 14-3-3β promoted HCC cell migration and invasion. Mechanistically, 14-3-3β augmented the expression of matrix metalloproteinase 2 (MMP2) and MMP9 through PI3K/Akt/NF-κB pathway, thereby enhancing the invasiveness of HCC cells. Furthermore, we show that a combinatory detection of 14-3-3β and p-Akt provides a better prognostic value for HCC patients. We have thus identified a novel pathway, PI3K/Akt/NF-κB/MMPs, which is activated by 14-3-3β in HCC malignancy.

## Materials and Methods

### Patients and samples

This study was reviewed and approved by the Clinical Research Ethics Committee of the General Hospital of Shenyang Military Area Command. Ninety-seven HCC patients who underwent curative resection in the General Hospital of Shenyang Military Area Command (Shenyang, China) from January2009 to March 2011 were randomly and retrospectively enrolled in this study in January 2013, and written informed consent was obtained from all participants. The researchers did not have access to the identifying information of the patients during or after date collection. HCC samples were retrieved to construct tissue microarray (TMA) to detect the expression of indicated proteins. All of the patients (100%, 97/97) had hepatitis B virus background. All patients were followed until December2014 with the longest follow-up up to 69 months. The time of the surgery was used to calculate the time to a given event. Overall survival (OS) and time to recurrence (TTR) were defined as previously described [19]. The diagnosis of tumor recurrence was based on cytologic/histologic evidence as well as the noninvasive diagnostic criteria for HCC used by the European Association for the Study of the Liver. Tumor stage was determined according to the 2002 International Union Against Cancer TNM classification system. Please see detailed clinicopathologic features in S2 Table. Microsatellite nodules that were defined as tumors adjacent to the border of the main tumor were only observed under the microscope. Microvascular invasion, defined as tumors spread to liver microvascualr but not to the main portal vein, was observed under the microscope. Early recurrence is defined as time to recurrence in less than 24 months. An additional 76 pairs of human HCC and peritumoral samples (collected between October 2011 and July 2012) were subjected to quantitative RT-PCR (64 pairs) and western blot analysis (12 pairs). Further, 34 pairs of HCC samples with portal vein tumor thrombi (PVTT) were obtained and used to perform quantitative RT-PCR (24 pairs) and western blot analysis (10 pairs).

### Cell culture and transfection

Human hepatoma cell lines Hep3B, HepG2 and SMMC-7721 were purchased from the Cell Bank of Chinese Academy of Sciences (Shanghai, China). Hep3B and HepG2 cells were cultured in MEM (Gibco, Carlsbad, CA, USA) supplemented with 10% FBS (Hyclone, Logan, UT, USA), while SMMC-7721 cells were cultured in RPMI-1640 (Gibco) supplemented with 10% FBS. CSQT-2 cell line, which was derived from PVTT of HCC and established by Dr Cheng’s lab [20], was a kind gift from Dr Cheng. CSQT-2 cells cultured in DMEM (Gibco) supplemented with 10% FBS. The cells were maintained at 37°C in a humidified atmosphere of 95% air and 5% CO_2_.

For overexpression, 14-3-3β cDNA was PCR-amplified and subcloned into the pcDNA3.1 expression vector (TaKaRa Clontech, Otsu, Japan). Hep3B and SMMC-7721 cells were transfected with 14-3-3β expression constructs or pcDNA3.1 vector using lipofectamin 2000 (Invitrogen, Carlsbad, CA, USA) according to the manufacturer’s instructions. For knocking down, short interfering RNAs (siRNAs) (Shanghai GenePharma Co.,Ltd, Shanghai, China) targeting two distinct sites on 14-3-3β mRNA were designed as follows: siRNA 786, 5’-GUCUUGGUCUGGCACUAAATTUUUAGUGCCAGACCAAGACTT-3’; siRNA 901, 5’-GCUGAAUGAAGAGUCUUAUTTAUAAGACUCUUCAUUCAGCTT-3’, and transfected into CSQT-2 cells using lipofectamin 2000 system. The cells were starved in serum-free medium for 1 h prior to transfection and the medium was replaced with fresh culture medium 6 h after transfection. At post-transfection 24 h, the cells were subjected to Western blot analysis or Transwell assay with or without the treatment with one of the following signaling inhibitors: LY294002 (Cell Signaling, Beverly, MA, USA), PDTC (Sigma-Aldrich, St. Louis, MO, USA), PD98059, SP600125 and SB203580 (all from Calbiochem, San Diego, CA, USA).

### Western blot

For total protein extraction, tissue samples were physically homogenized and lysed with RIPA lysis buffer (Beyotime, Haimen, China). The transfected cells were incubated with the indicated inhibitors for 5 h and then lysed with NP-40 lysis buffer (Beyotime) to extract total proteins or were subjected to nuclear protein extraction with a Nuclear and Cytoplasmic Protein Extraction Kit (Beyotime) following the manufacturer’s instructions. A total of 20 μg proteins from each sample were separated by SDS-PAGE, and transferred onto PVDF membranes (Millipore, Bedford, MA, USA). The membrane was incubated with a specific primary antibody against the protein of interest overnight at 4°C, followed by incubation with a horseradish peroxidase (HRP)-conjugated IgG secondary antibody (Beyotime) for 1 h at room temperature. Anti-14-3-3β antibody was purchased from ABGENT (San Diego, CA, USA); anti-p-IκB antibody was purchased from Bioss (Beijing, China); primary antibodies against MMP2, MMP9, P38, p-P38, JNK, p-JNK, ERK1/2, P-ERK1/2, Akt, p-Akt, P65, E-cadherin, N-cadherin and Vimentin were purchased from Wanleibio (Shenyang, China). The detailed information about the antibodies is shown in S1 Table. The immune complexes were finally visualized using the ECL system (7SeaPharmTech, Shanghai, China). To verify equal protein loading and transfer, membranes were stripped with the stripping buffer (Beyotime) and re-probed with anti-β-actin antibody (Wanleibio) for total proteins or with anti-Lamin A antibody (Santa Cruz, Dallas, TX, USA) for nuclear proteins.

### Real-time polymerase chain reaction (PCR)

Total RNA was isolated with a RNAsimple total RNA kit (TIANGEN, Beijing, China), followed by reverse transcription using Super M-MLV reverse transcriptase (BioTeke, Beijing, China). Quantitative real-time PCR was performed using SYBR Green Master Mix (Solarbio, Beijing, China) in an Exicycler^TM^ 96 quantitative fluorescence analyzer (Bioneer, Daejeon, Korea), with the specific primers: 14-3-3β, Forward: 5’-CACGGGAATGACAATGGATAA-3’, Reverse: 5’-CTCAATGCTGGAGATGACACG-3’; β-actin, Forward: 5’-CTTAGTTGCGTTACACCCTTTCTTG-3’, Reverse: 5’-CTGTCACCTTCACCGTTCCAGTTT-3’.

### Immunohistochemistry/Tissue microarray (TMA)

Matched pairs of primary HCC tissues and adjacent liver samples were used for the construction of a TMA (in collaboration with the Shanghai Biochip Company, Shanghai, China). The TMA construction was performed as previously described [21]. Immunostaining was performed on TMA slides following the routine protocol. Following antigen retrieval, the samples were incubated with 3% H_2_O_2_, blocked with goat serum and probed with anti-14-3-3β antibody (1:50;ABGENT) overnight at 4°C, followed by serial incubations with HRP-conjugated secondary antibody and diaminobezidine (Sigma-Aldrich) for chromogenic reactions. The samples were also probed for p-Akt, using anti-p-Akt antibody (1:50, Wanleibio), followed by reactions with alkaline phosphatase-conjugated secondary antibody and the chromogen substrate BCIP/NBT (Beyotime). Cell nuclei were counter-stained with hematoxylin.

The TMA slides were scanned with an Aperio ScanScope GL and assessed by the Aperio ImageScope software (Aperio Technologies, Vista, CA). Scoring of the TMA samples was based on the percentage of positively stained cells and the staining intensity. The scores equal to or above the median of all the values were defined as high, while the scores below the median were defined as low.

### Transwell assay

Cell migration ability was assessed by the Transwell assay with uncoated upper chambers (Corning, New York, USA) [22], whereas cell invasion was examined by the Transwell assay with the upper chamber pre-coated with phenol red-free Matrigel (BD Biosciences, San Jose, CA, USA). The cells were pre-treated with 5 μM mitomycin-C for 2 h to inhibit proliferation. 2×10^4^ cells resuspended in 200 μl serum-free medium were plated in the Transwell upper chamber, and allowed to migrate toward the bottom wells containing 800 μl culture medium supplemented with 20% FBS for 24 h in a 37°C incubator. Thereafter, cells remaining on the top surface of the Transwell microporous membrane were removed, and the cells on the bottom surface of the membrane were fixed with paraformaldehyde and stained with 0.1% crystal violate. Under a 200× inverted microscope, five fields on each membrane were randomly selected, and the mean number of invading cells was calculated.

### Statistical analysis

The data were analyzed using the SPSS version 2.0 (IBM, Chicago, IL, USA). Wilcoxon signed-rank test was used to analyze the differences between primary tumors and matched nontumorous tissues or PVTTs. The Student’s *t*-test was used to compare differences between two groups. Kaplan-Meier curves of cumulative survival and recurrence were plotted, and the differences were analyzed by the log-rank test. Factors that influence post-surgical survival and recurrence were analyzed by multivariate analysis using Cox proportional hazard regression models, and the results are reported as hazard ratios (HR) with their 95% confidence intervals (CI). The correlation coefficients between the levels of 14-3-3β and p-Akt in the primary tumors were calculated by the Spearman correlation test. All *P* values were two-tailed, and the differences with *p*<0.05 were considered statistically significant.

## Results

### Expression of 14-3-3β is up-regulated in human HCC tissues and invasive HCC cell lines

**Fig 1:**
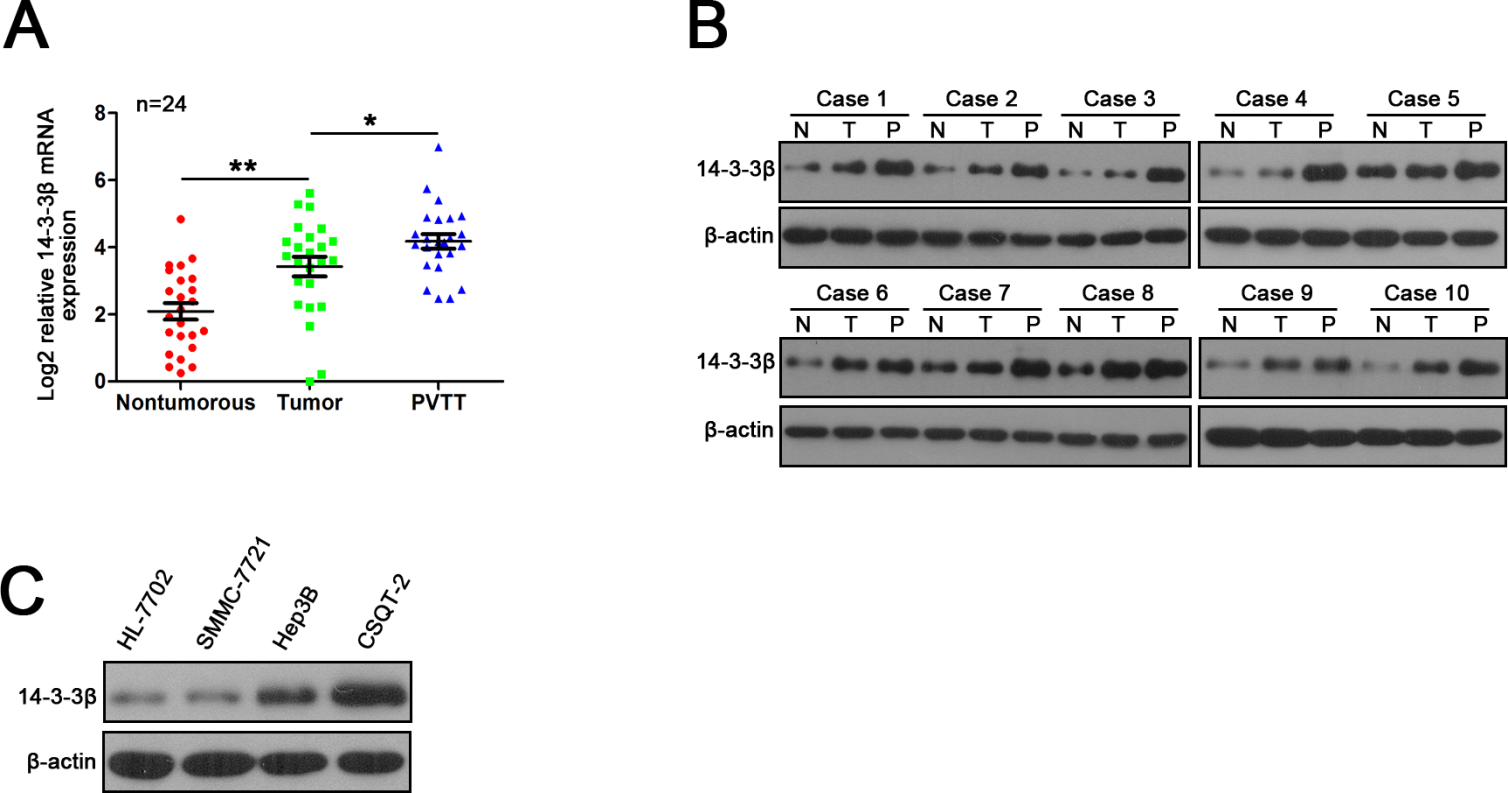
Increased expression of 14-3-3β in primary HCC tumors, PVTTs and HCC cell lines. (**A)** Real-time PCR and **(B)** western blot analysis of mRNA and protein levels of 14-3-3β in primary tumors (T), matched adjacent nontumorous liver tissues (N) and PVTTs (P) in HCC patients. (**C)** Western blot analysis of 14-3-3β expression in several HCC cell lines. β-actin was used as the internal control for all analysis. *p<0.05; **p<0.01.

We first examined 14-3-3β expressionin paired HCC tissues. 14-3-3β transcripts were significantly increased in the primary tumor tissue of HCC relative to the paired nontumorous tissue in 64 HCC patients, and the elevation of 14-3-3β expression was further confirmed by western blot assay (S1A & S1B Fig). We next performed Immunohistochemical (IHC) analysis of 14-3-3β expression using HCC TMA containing 97 paired HCC samples. As shown in S1C Fig, the staining density of 14-3-3β protein in the tumor tissues was stronger than that in the nontumorous tissues (average staining density of 14-3-3β protein: 0.359±0.068 vs 0.267±0.041, *p*<0.001). Portal vein tumour thrombus (PVTT), arising from the invasion of HCC cells into the portal vein, serves as a poor prognostic factor of metastasis. The mRNA and protein levels of 14-3-3β were significantly increased in the PVTT compared with the matched primary tumor and the nontumorous tissue (Fig 1A & 1B). Furthermore, we examined the expression of 14-3-3β protein in three HCC cell lines (SMMC-7721, Hep3B and CSQT-2) and one normal liver cell line (HL-7702) by western blot analysis. As shown in Fig 1C, consistent with the results from the primary tissues, a higher level of 14-3-3β protein was observed in the more aggressive HCC cell line (CSQT-2) than that in the normal liver cells (HL-7702) and the less malignant HCC cell lines (SMMC-7721 and Hep3B). All of these results suggested a potential role of 14-3-3β in HCC metastasis.

### 14-3-3β overexpression predicts poor prognosis of HCC

**Fig 2:**
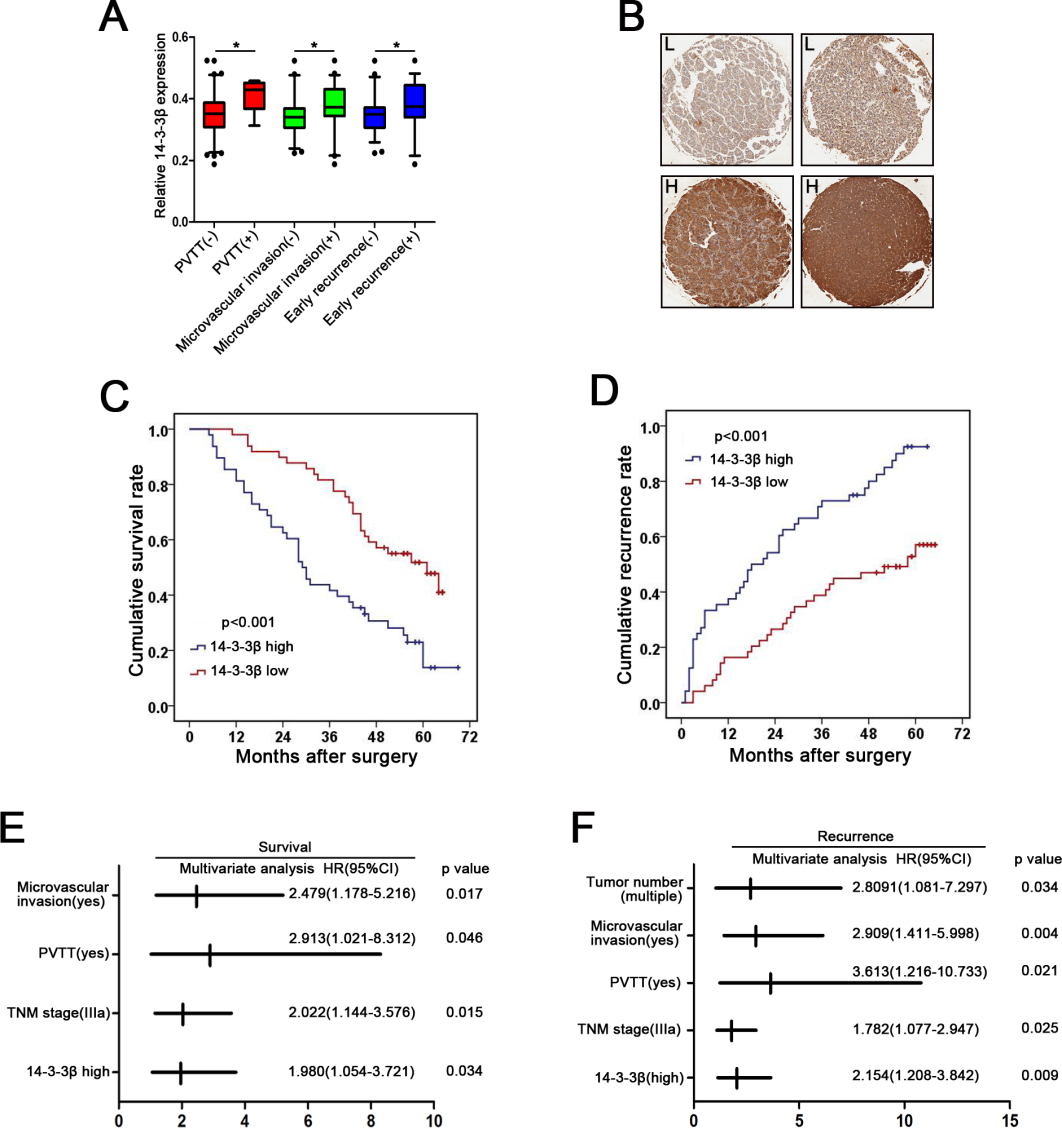
High expression of 14-3-3β in primary HCC tumors is associated with poor survival and high recurrence of HCC patients after surgical resection. **(A)** Relative expression levels of 14-3-3β in the primary HCC tumors grouped by the presence or absence of different clinical parameters, with the means and 95% confidence intervals. *p<0.05. **(B)** Immunohistochemical examination of 14-3-3β in primary HCC tumors. H, high; L, low. **(C, D)** Kaplan-Meier curves of cumulative post-surgical survival and recurrence in HCC patients with high or low level of 14-3-3β. **(E, F)** Multivariate analysis of parameters associated with the unfavorable post-surgical survival and recurrence. HR, hazard ratio; CI, confidence interval.

To further investigate the clinical significance of 14-3-3β in the development and progression of HCC, all 97 HCC patients were divided into two groups based on the overall expression level of 14-3-3β: a high 14-3-3β expression group (n = 49) and a low 14-3-3β expression group (n = 48) (S2 Table). As shown in Fig 2A and Table 1, overexpression of 14-3-3β was significantly correlated with PVTT (*p*<0.05), microvascular invasion (*p*<0.05) and early recurrence (*p*<0.05) in HCC patients. More importantly, the patients with high 14-3-3β expression (Fig 2B, S3 Table) exhibited worse overall survival (OS, median OS time were 29.5 and 53 months, respectively, difference = 23.5 months, *p*<0.001) and shorter time to recurrence (TTR, median TTR were 19.5 and 50 months, respectively, difference = 30.5 months, *p*<0.001) (Fig 2C & 2D, S4 Table). Consistently, the 3-year and 5-year OS rates after surgery were much lower in 14-3-3β-high group than that in 14-3-3β-low group, whereas the TTR rate was much higher in14-3-3β-high group than that in 14-3-3β-low group (S5 Table). Furthermore, the multivariate analysis indicated 14-3-3β expression level, together with microvascular invasion, PVTT and TNM stage, was an independent risk factor for both OS and TTR for HCC patients (Fig 2E & 2F, Table 2). The 14-3-3β-high group also displayed a shorter OS time and a higher risk of tumor recurrence (OS: hazard ratio (HR) = 1.980, 95% confidential interval (CI) = 1.054–3.721, *p* = 0.034; TTR: HR = 2.154, 95% CI = 1.208–3.842, *p* = 0.009). Taken together, 14-3-3β could serve as a valuable predicting factor for poor survival and recurrence of HCC patients.

**Table 1:** Relationship between 14-3-3β expression and clinicopathologic features of HCC patients.

		Relative 14-3-3β expression		*P* value
Features		Low	High	
Sex	male	43	40	0.536
	female	6	8	
Age (yrs)	≤60	41	41	0.812
	>60	8	7	
HBeAg	positive	13	14	0.772
	negative	36	34	
Liver cirrhosis	yes	38	40	0.473
	no	11	8	
AFP (ng/ml)	≤20	21	18	0.591
	>20	28	30	
ALT (U/L)	≤45	29	26	0.618
	>45	20	22	
Tumor size (cm)	≤3	32	28	0.480
	>3	17	20	
Tumor number	single	42	47	0.069
	multiple	7	1	
Encapsulation (complete)	yes	9	14	0.211
	no	40	34	
Microvascular invasion	yes	16	28	0.011
	no	33	20	
PVTT	yes	1	8	0.033
	no	48	40	
Tumor differentiation	II	19	10	0.054
	III	30	38	
TNM stage	I	39	29	0.111
	II	5	8	
	IIIa	5	11	
Early recurrence (≤24 months)	yes	13	26	0.006
	no	36	22	

**Note:** Statistical analysis was done by Chi-square test. Abbreviations: HBeAg, hepatitis B e antigen; AFP, alpha fetoprotein; ALT, alanine aminotransferase; PVTT, portal vein tumor thrombus.

**Table 2:** Univariate and multivariate analysis of factors associated with survival and recurrence of HCC patients.

Features	Overall survival				Time to recurrence			
	Univariate	Multivariate			Univariate	Multivariate		
	*P* value	Hazard ratio	95% CI	*P* value	*P* value	Hazard ratio	95% CI	*P* value
Sex (male *vs*. female)	0.143			NA	0.055			NA
Age (≤60 *vs*. >60 years old)	0.251			NA	0.340			NA
HBeAg (positive *vs*. negative)	0.413			NA	0.393			NA
Liver cirrhosis (yes *vs*. no)	0.712			NA	0.693			NA
AFP (≤20 *vs*. >20 ng/ml)	0.016			NS	0.01			NS
ALT (≤45 *vs*. >45 U/L)	0.064			NA	0.081			NA
Tumor siz e(≤3 *vs*. >3 cm)	<0.001			NS	<0.001			NS
Tumor number (single *vs*. multiple)	0.003			NS	<0.001	2.691	1.035–6.999	0.042
Encapsulation (yes *vs*. no complete)	0.902			NA	0.583			NA
Microvascular invasion (yes *vs*. no)	<0.001	2.479	1.178–5.216	0.017	<0.001	2.965	1.433–6.135	0.003
PVTT (yes *vs*. no)	<0.001	2.913	1.021–8.312	0.046	<0.001	3.644	1.230–10.795	0.020
Tumor differentiation (II *vs*. III)	0.005			NS	0.004			NS
TNM stage (I *vs*. II *vs*. IIIa)	<0.001	2.022	1.144–3.576	0.015	<0.001	1.792	1.085–2.961	0.023
14-3-3β expression (low *vs*. high)	<0.001	1.980	1.054–3.721	0.034	<0.001	2.043	1.140–3.661	0.016

**Note:** Univariate analysis was performed using the Kaplan-Meier method. Multivariate analyses were done by Cox proportional hazard regression models. **Abbreviations:** CI, confidence interval; HBeAg, hepatitis B e antigen; AFP, alpha fetoprotein; ALT, alanine aminotransferase; PVTT, portal vein tumor thrombus; NA, not adopted; NS, not significant.

### 14-3-3β promotes HCC cell migration and invasion

**Fig 3:**
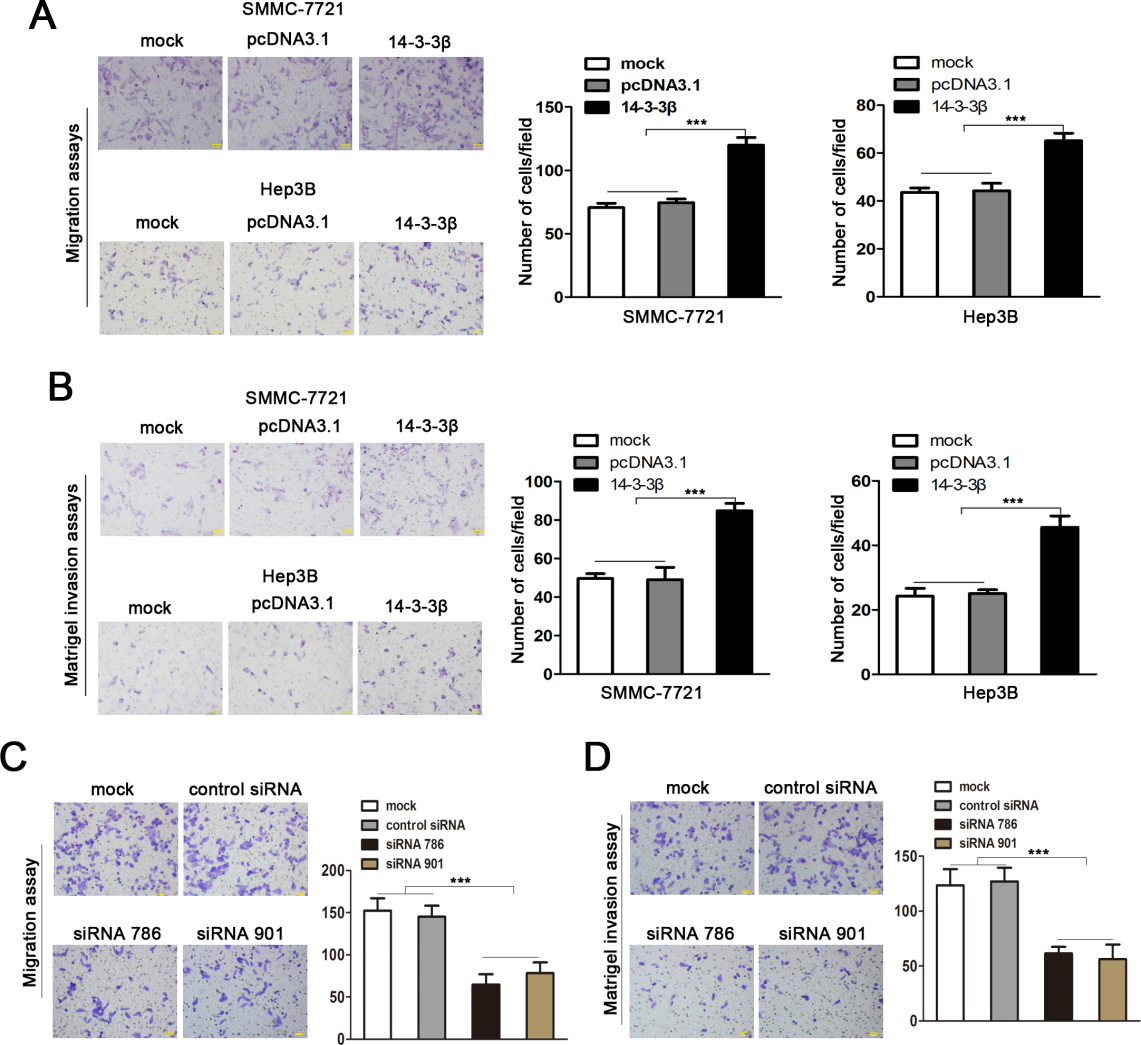
14-3-3β promotes migration and invasion of HCC cells. **(A)** Matrigel-free Transwell assay was conducted to assess the migration ability of SMCC-7721 and Hep3B cells that were transfected with 14-3-3β expression construct or the empty vector pcDNA3.1. (B) Matrigel-based Transwell assay was performed to examine the invasiveness of SMCC-7721 and Hep3B with exogenous expression of 14-3-3β. **(C)** Matrigel-free migration assay and **(D)** Matrigel-based invasion assay were performed in CSQT-2 cells with and without expression of 14-3-3β-specific siRNA. This figure shows the representative images and the statistical analysis of three independent experiments. Values are expressed as the mean ± standard deviation. ***p<0.001.

Based on the observation that 14-3-3β was highly expressed in the metastatic HCC primary tumors and PVTTs, we further investigated the role of 14-3-3β in HCC migration and invasion. We constructed a pcDNA3.1-14-3-3β plasmid containing the 14-3-3β open reading frame. Then, pcDNA3.1-14-3-3β was transfected into SMMC-7721 and Hep3B cells that displayed a relative lower 14-3-3β expression among the HCC cell lines tested, in order to overexpress 14-3-3β in the HCC cells (SMMC-7721/14-3-3β and Hep3B/14-3-3β), while pcDNA3.1 was used as a control (SMMC-7721/pcDNA3.1 and Hep3B/pcDNA3.1) (S2A Fig). The migration and Matrigel-based invasion assays with these 14-3-3β overexpressing cells showed that exogenous expression of 14-3-3β significantly enhanced the ability of migration and invasion of HCC cells (Fig 3A & 3B). To confirm that14-3-3β affects the metastatic potential of HCC cells, we next examined whether the migration and invasion of HCC cells were affected by14-3-3β-specific siRNAs. Two chemically synthesized siRNAs were transfected into CSQT-2 cells that displayed the highest 14-3-3β expression among the HCC cell lines tested, in order to silencing the expression of 14-3-3β (S2B Fig). 14-3-3β silencing strongly inhibited the capacity of migration and invasion of the transfected cells as compared with the control cells (Fig 3C & 3D). Therefore, the above results indicated that 14-3-3β enhanced the metastatic potential of HCC cells.

### 14-3-3β modulates the expression of MMP2 and MMP9 via PI3K/Akt/NF-κB signaling pathway

**Fig 4:**
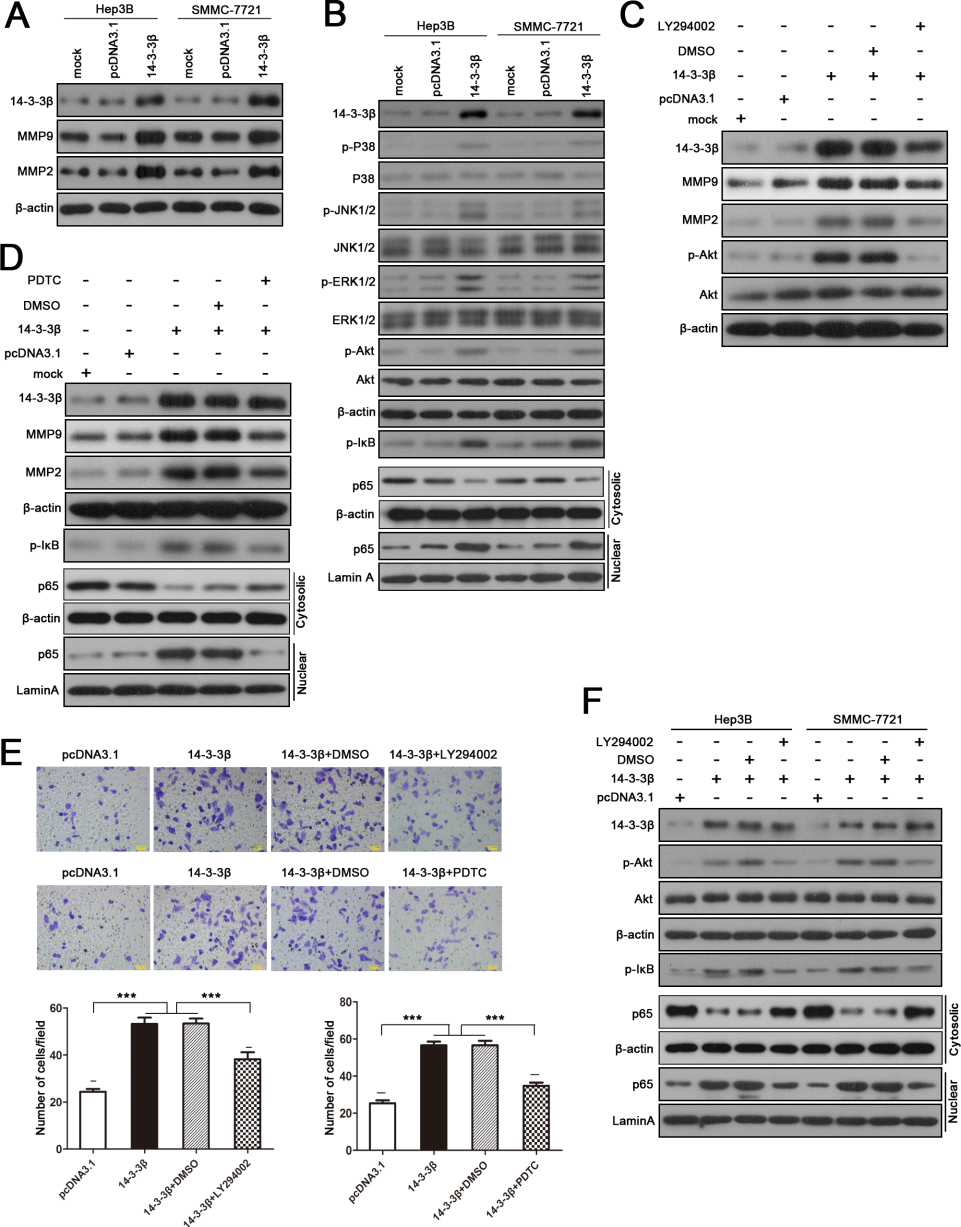
14-3-3β-regulated expression of MMP2 and MMP9 is dependent on PI3K/Akt/NF-κB signaling pathway. **(A)** Expression of 14-3-3β, MMP2 and MMP9 in 14-3-3β-transfected SMCC-7721 and Hep3B cells. **(B)** Western blot analysis of phosphorylation status of various signaling proteins and the level of nuclear p65 in 14-3-3β-overexpressing HCC cells. β-actin was used as the internal control for total or cytosolic proteins, while Lamin A served as the internal control for nuclear proteins. The 14-3-3β-transfected Hep3B cells were treated with **(C)** LY294002 (20 μM), the inhibitor of PI3K/Akt signaling, or **(D)** PDTC (10 μM), the inhibitor of NF-κB signaling, for 5 h, followed by western blot analysis of the expression of MMP2 and MMP9. **(E)** The invasiveness of 14-3-3β-overexpressing cells with and without LY294002 (20 μM) or PDTC (10 μM) treatment was assessed by Matrigel-based Transwell assay. Values are expressed as the mean ± standard deviation of three independent experiments. ***p<0.001. **(F)** Western blot analysis was performed to determine the activation status of p-Akt and NF-κB in 14-3-3β-overexpressing cells after treating the cells with LY294002 (20 μM) for 5 h.

To explore the underlying molecular mechanism for 14-3-3β-mediated HCC invasion, we first examined whether 14-3-3β is involved in the regulation of epithelial-mesenchymal transition (EMT) in HCC, which is considered to be a key process during tumor metastasis. As shown in S3 Fig, 14-3-3β showed no significant effect on the expression of EMT-related genes, such as E-cadherin, N-cadherin and Vimentin, indicating that14-3-3β-mediated HCC metastasis is EMT independent. Since MMPs are another key mediators in cell invasion, we next investigated whether 14-3-3β affected MMPs expression in HCC cells. Importantly, we found that 14-3-3β up-regulated MMP2 and MMP9 expression level in HCC cells (Fig 4A).

Previous studies have reported that 14-3-3 proteins play an important role in the regulation of several cancer-related signaling pathways. As expected, we found that increased 14-3-3β expression in HCC cells induced marked phosphorylation of Akt, mitogen-activated protein kinase (MAPK, also known as p38), extracellular regulated protein kinase 1/2 (ERK1/2), and Jun-N-terminal kinase 1/2 (JNK 1/2) (Fig 4B). Moreover, 14-3-3β overexpression also resulted in activation of NF-κB signaling pathway which was indicated by phosphorylation of inhibitor of NF-κB (IκB) and nuclear translocation of p65 (Fig 4B). We further noted that 14-3-3β-induced up-regulation of MMP2 and MMP9 could be abolished by the PI3K inhibitor LY294002 and the NF-κB inhibitor PDTC (Fig 4C & 4D), but it was not affected by blocking MAPK/ERK1, P38 or JNK signaling pathways (S4 Fig). Moreover, the invasiveness of 14-3-3β-overexpressing cells was significantly attenuated when PI3K/Akt or NF-κB signaling pathway was inhibited (Fig 4E). Interestingly, we found that inhibiting PI3K/Akt signaling in 14-3-3β-overexpressing cells by LY294002 suppressed the activation of NF-κB signaling pathway (Fig 4F), implying 14-3-3β promotes HCC cells migration and invasion via PI3K/Akt/NF-κB pathway.

### The combination of 14-3-3β and p-Akt provides a better prognostic value for HCC patients

**Fig 5:**
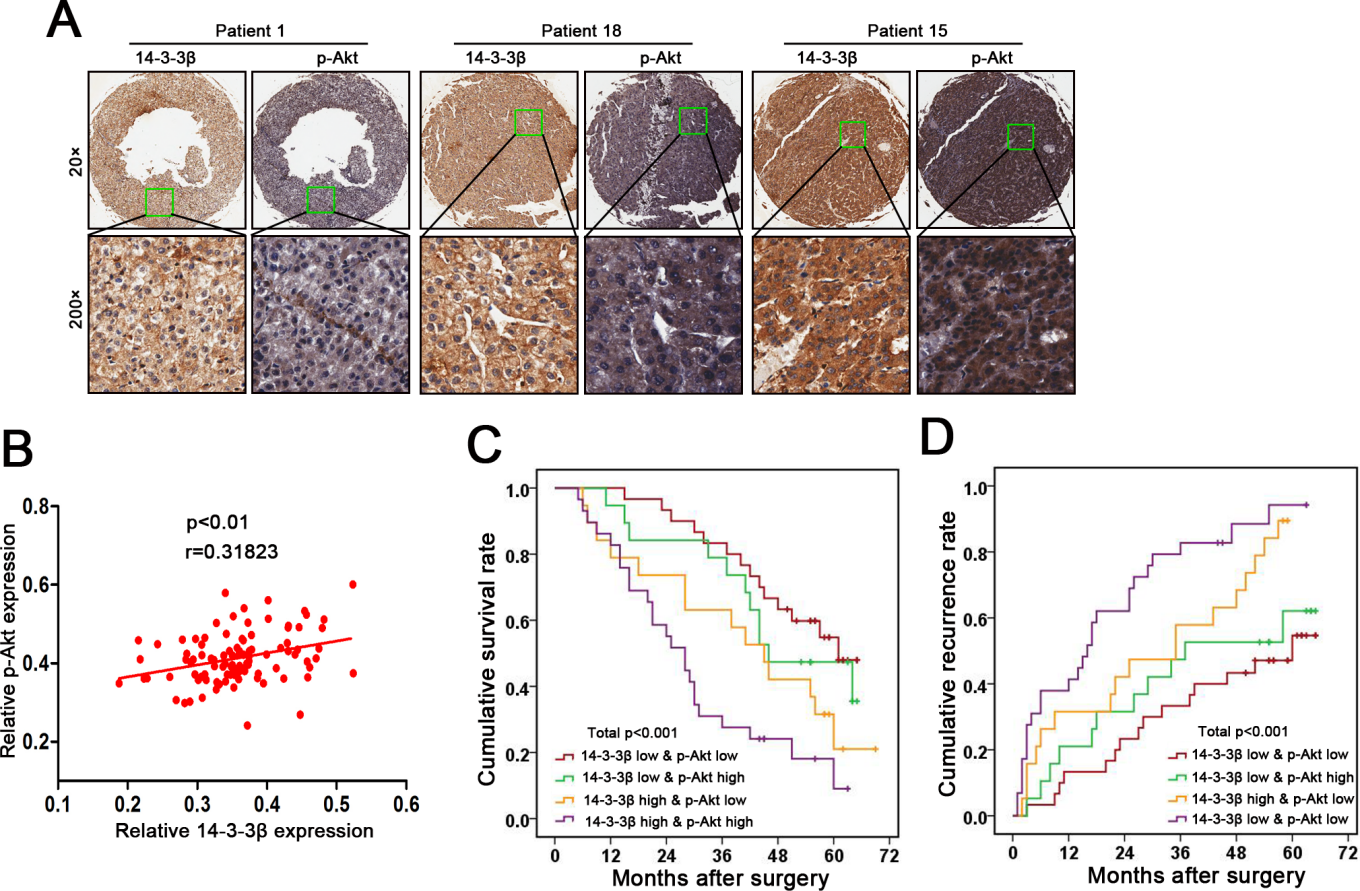
Correlated elevation of 14-3-3β and p-Akt in primary HCC tumors predicts a worse post-surgical outcome in HCC patients. **(A)** The levels of 14-3-3β and p-Akt in the primary HCC tumors were examined by tissue microarray. **(B)** The correlation of the levels between 14-3-3β and p-Akt in HCC tumors was analyzed using the Spearman correlation test. Kaplan-Meier curves of **(C)** cumulative post-surgical survival and **(D)** cumulative post-surgical recurrence in HCC patients grouped by the high or low levels of 14-3-3β and p-Akt.

As shown in Fig 5A & 5B, tissues microarray analysis of 97 patients’ specimens revealed a significant positive correlation between 14-3-3β and p-Akt expression levels (*p*<0.01), which further supports the notion that 14-3-3β activates Akt in HCC. Elevation of either 14-3-3β or p-Akt in HCC tissues predicted a poor prognosis of the patient (S5 Fig). The patients bearing HCC with above-medium levels of both 14-3-3β and p-Akt suffered exacerbated adverse clinical outcomes compared with those with below-medium levels of both 14-3-3β and p-Akt (OS, *p*<0.001; TTR, *p*<0.001; Fig 5C & 5D). Collectively, our results suggest that combinatory evaluation of 14-3-3β expression and p-Akt signal is a power predictor of prognosis in HCC patients. In addition, the results reinforce the model of 14-3-3β-mediated activation of PI3K/Akt/NF-κB pathway, which accounts for the up-regulation of MMP2 and MMP9 in HCC cells and thus promotes metastasis.

## Discussion

Emerging evidences have been shown to support that 14-3-3 plays a critical role in the pathogenesis and metastasis of cancers. For example, 14-3-3ζ was found to interact with ErbB2 to regulate several important metastasis mediators including E-cadherin, fork head box protein M1 (FOXM1) and β-catenin, such as to promote breast cancer metastasis [23]. In addition, 14-3-3ε has been demonstrated to promote EMT by inducing Zeb-1 and Snail expression, thereby promoting cell migration and invasion of HCC [24]. Liu et al. also identified that aberrant expression of 14-3-3β is associated with extrahepatic metastasis and worsened survival in HCC patients [16]. In the current study, consistent with Liu et al.’s findings, our results indicate that high 14-3-3β expression in primary HCC tissues is associated with intraheaptic metastasis and significantly worsened clinical outcome. Furthermore, we demonstrate that the pro-metastatic activity of 14-3-3β is most likely attributed to 14-3-3β-mediated activation of PI3K/Akt/NF-κB pathway, which consequently up-regulates the expression of MMP2 and MMP9 in HCC cells.

An earlier study reported that Snail can selectively interacted with14-3-3γ, 14-3-3ε, 14-3-3τ, 14-3-3η and 14-3-3β in MCF breast cancer cells and 293T cells, and such interaction represses the expression of E-cadherin and further triggers EMT [25]. However, in our study, elevated 14-3-3β expression did not affect the expression of the key EMT-related markers in HCC cells, implying that14-3-3β-mediated HCC cell invasion and metastasis is EMT independent. The discrepancy between our results and the previous study is probably due to the differential functions that 14-3-3 exerts in different cell types.

The expression level of MMPs is implicated to be correlated with the metastatic ability of cancer cells. Particularly, abnormal expression of MMP2 and MMP9 is often detected in solider tumor tissues and is associated with tumor metastasis in many cancers including HCC [26]. In this study, we demonstrated that 14-3-3β overexpression in HCC cell line enhanced cell migration and invasion, accompanied by up-regulated expression of MMP2 and MMP9, suggesting that 14-3-3β-regulated expression of MMP2 and MMP9 is important for HCC invasion and metastasis. Previous functional studies of 14-3-3β mainly focused on its role in cell proliferation and tumor growth [16–18], and our study supports Liu’s work [16] by demonstrating the important role of 14-3-3β in tumor invasion and metastasis. Furthermore, by using specific inhibitors of various cancer-related signaling pathways, we showed that 14-3-3β-induced up-regulation of MMP2 and MMP9 was dependent on PI3K/Akt/NF-κB signaling pathway. Liu et al. previously demonstrated that 14-3-3β-promoted hepatic tumor cell migration and invasion could be suppressed by inhibiting MEK/ERK (MAPK) signaling pathway [16], which, however in our study, did not show any effect on 14-3-3β-induced up-regulation of MMP2 and MMP9.

It is generally believed that 14-3-3 proteins bind to a wide range of signaling proteins, especially kinases, facilitate their phosphorylation and protect their phosphorylated sites from dephosphorylation, thereby prolonging their activation status. Thus, enhanced activation of various signaling pathways was observed in 14-3-3β-overexpressing cells in our study. Raf proteins, which are the key effectors of Ras GTPase that initiates the activation of MEK/ERK cascade, were among the first identified 14-3-3 targets [27]. In addition to MEK/ERK cascade, the involvement of which in 14-3-3β-regulated tumor cell invasion has been described previously [16], we identified that PI3K/Akt and NF-κB signaling pathways also played a role in 14-3-3β-regulated invasiveness of HCC cells. Akt is a key kinase that generates 14-3-3 binding sites on a diverse array of target proteins [28], thus inhibition of PI3K/Akt signaling abolishes the interactions of 14-3-3 proteins with various targets and therefore abate their functions, in this case, transactivation of MMP2/9 and other components that are required for cell invasion. In this study, we found that PI3K/Akt signaling also contributed to the activation of NF-κB pathway, and the crosstalk between them might be mediated via the signaling transducers such as IκB kinase (IKK) [29].

Consistent with a previous study [16], our clinical data indicated that increased 14-3-3β expression was associated with poor survival and high recurrence in HCC patients after surgical resection, supporting the clinical value of 14-3-3β as a prognostic marker in HCC. Further, with the molecular basis of the participation of PI3K/Akt pathway in 14-3-3β-regulated tumor cell invasion as well as the tissue microarray data, we show that 14-3-3β in combination with p-Akt, the level of which is positively correlated with the expression of 14-3-3β in the primary HCC tumors, can better predict post-surgical outcome of HCC patients.

In conclusion, our study provides clinical and experimental evidence to support that 14-3-3β plays an important role in HCC invasion and metastasis. Our results also suggest that 14-3-3β promotes HCC cell invasion by up-regulating MMP2 and MMP9 via PI3K/Akt/NF-κB signaling pathway. In addition, we propose a combinatory detection of 14-3-3β and p-Akt for more accurate assessment of prognosis of HCC patients.

## Supporting Information

S1 Fig Increased expression of 14-3-3β in primary tumors of HCC patients. **(A)** Real-time PCR analysis of mRNA levels and **(B)** western blot analysis of protein levels of 14-3-3β in the primary HCC tumors (T) versus the adjacent nontumorous tissues (N) were performed using β-actin as the internal control. **(C)** Representative images of tissue microarray for 14-3-3β in the primary tumors and the matched nontumorous liver tissues, and the statistical analysis of tissue microarray results from 97 HCC patients. ***p<0.001.
(TIF)

S2 Fig Overexpression and knocking-down of 14-3-3β in HCC cells. **(A)** SMMC-7721 and Hep3B cells were transfected with 14-3-3β expression vector or pcDNA3.1 empty vector, followed by western blot analysis of 14-3-3β expression. **(B)** CSQT-2 cells were transfected with 14-3-3β-specific siRNA (siRNA 786 or siRNA 901) or the control siRNA, followed by western blot analysis of 14-3-3β expression.
(TIF)

S3 Fig Expression of EMT-related proteins in 14-3-3β-overexpressing cells. SMMC-7721 and Hep3B cells were transfected with 14-3-3β expression vector or pcDNA3.1 empty vector. The protein levels of E-cadherin, V-cadherin and Vimentin were analyzed by western blotting 24 h after transfection.
(TIF)

S4 Fig 14-3-3β-induced upregulation of MMP2 and MMP9 is independent of MEK/ERK or JNK signaling pathways. 14-3-3β- or pcDNA3.1-transfected Hep3B cells were treated with **(A)** 20 μM PD98059, a MEK inhibitor, **(B)** 20 μM SP600125, a JNK inhibitor, or **(C)** 10 μM SB203580, a P38/ERK2 inhibitor, for 5 h, and subjected to western blot analysis of the inhibitory effect on signal transduction and the expression of MMP2 and MMP9.
(TIF)

S5 Fig Enhanced activation of Akt worsens post-surgical survival and promotes recurrence in HCC patients. **(A)** Cumulative post-surgical survival rate and **(B)** cumulative post-surgical recurrence rate were analyzed by Kaplan-Meier curves in HCC patients with high or low levels of p-Akt.
(TIF)

S1 Table Information about the antibodies used in Western blotting. (DOCX)

S2 Table Clinicopathologic features of HCC patients. (DOCX)

S3 Table Density of intratumoral/peritumoral 14-3-3β staining. (DOCX)

S4 Table Relationship between intratumoral 14-3-3β expression and survival time. (DOCX)

S5 Table Relationship between intratumoral 14-3-3β expression and survival or recurrence rate. (DOCX)
