# Supplementary figures and images for: 14-3-3β Promotes Migration and Invasion of Human Hepatocellular Carcinoma Cells by Modulating Expression of MMP2 and MMP9 through PI3K/Akt/NF-κB Pathway
Source: PLoS One. 2016 Jan 5;11(1):e0146070. doi: 10.1371/journal.pone.0146070 (PMC4711775; doi:10.1371/journal.pone.0146070)

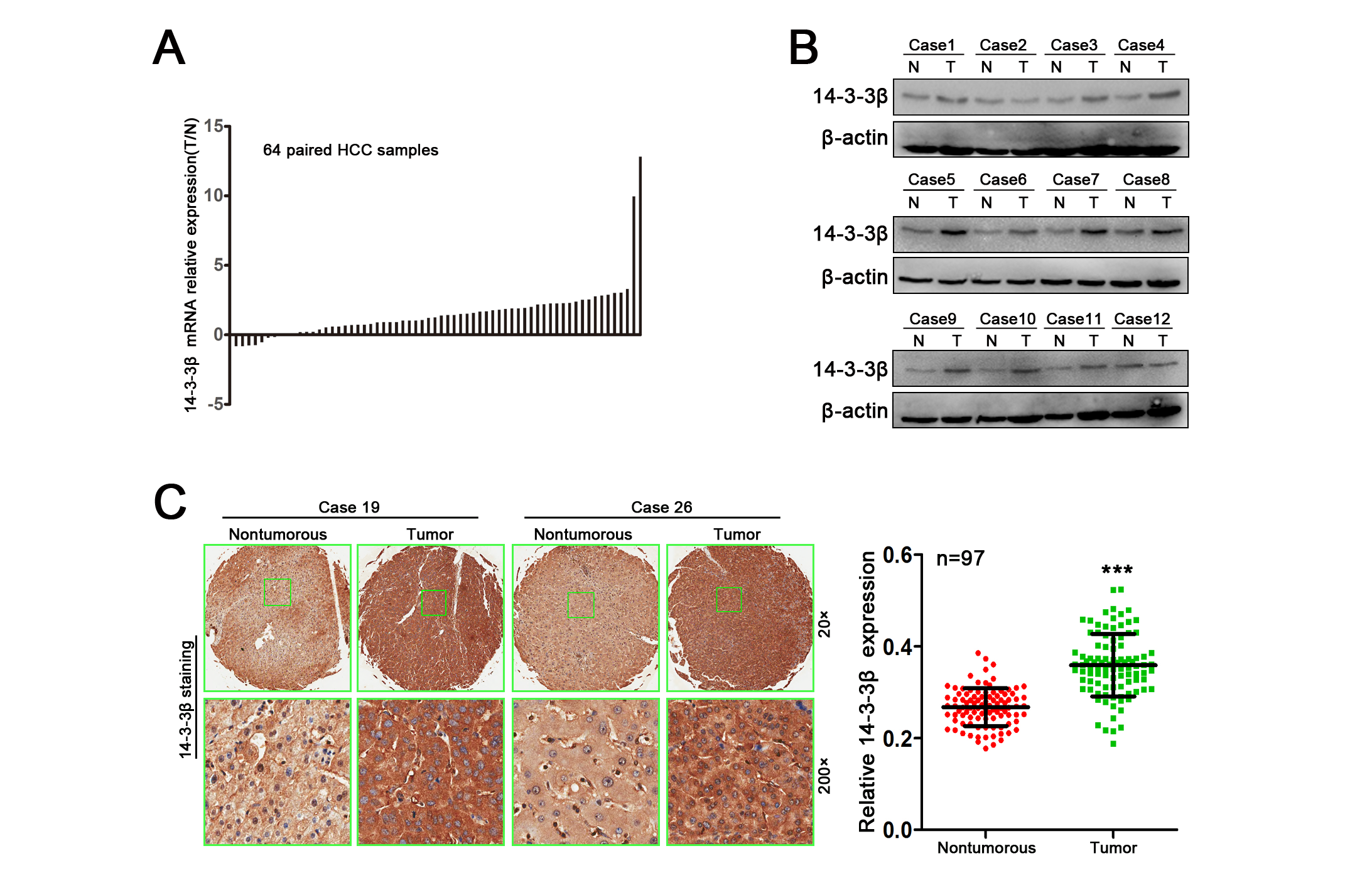

Supplement: S1 Fig — (A) Real-time PCR analysis of mRNA levels and (B) western blot analysis of protein levels of 14-3-3β in the primary HCC tumors (T) versus the adjacent nontumorous tissues (N) were performed using β-actin as the internal control. (C) Representative images of tissue microarray for 14-3-3β in the primary tumors and the matched nontumorous liver tissues, and the statistical analysis of tissue microarray results from 97 HCC patients. ***p<0.001. (TIF) [file pone.0146070.s001.tif]

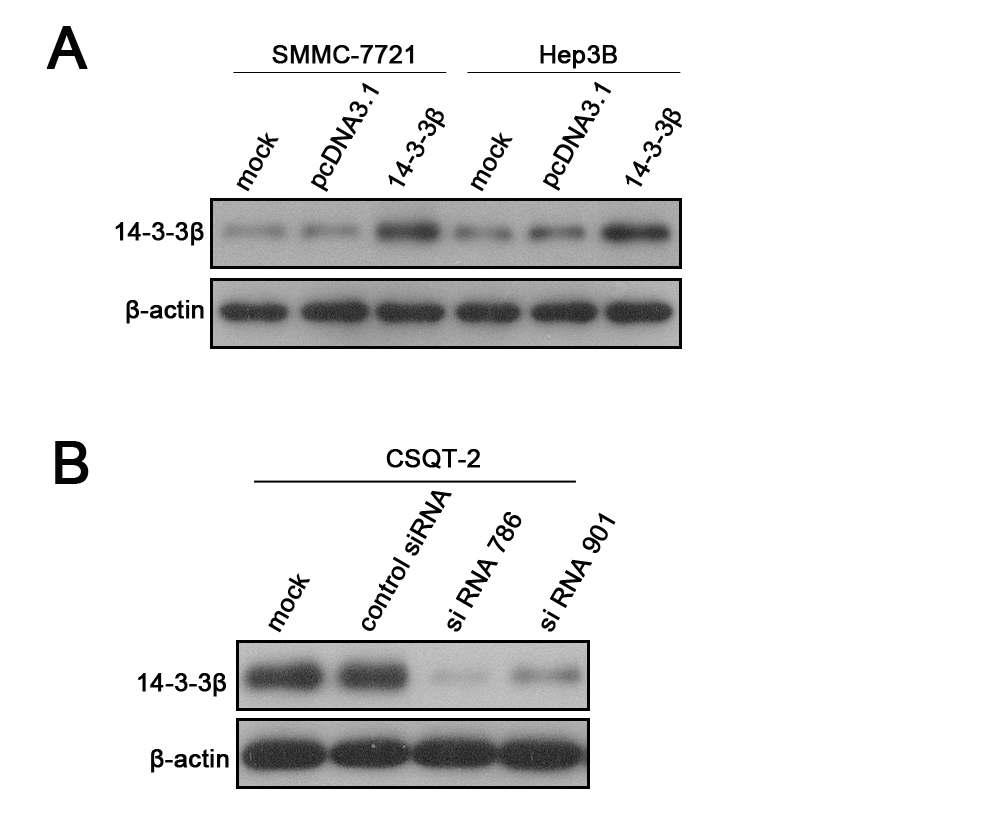

Supplement: S2 Fig — (A) SMMC-7721 and Hep3B cells were transfected with 14-3-3β expression vector or pcDNA3.1 empty vector, followed by western blot analysis of 14-3-3β expression. (B) CSQT-2 cells were transfected with 14-3-3β-specific siRNA (siRNA 786 or siRNA 901) or the control siRNA, followed by western blot analysis of 14-3-3β expression. (TIF) [file pone.0146070.s002.tif]

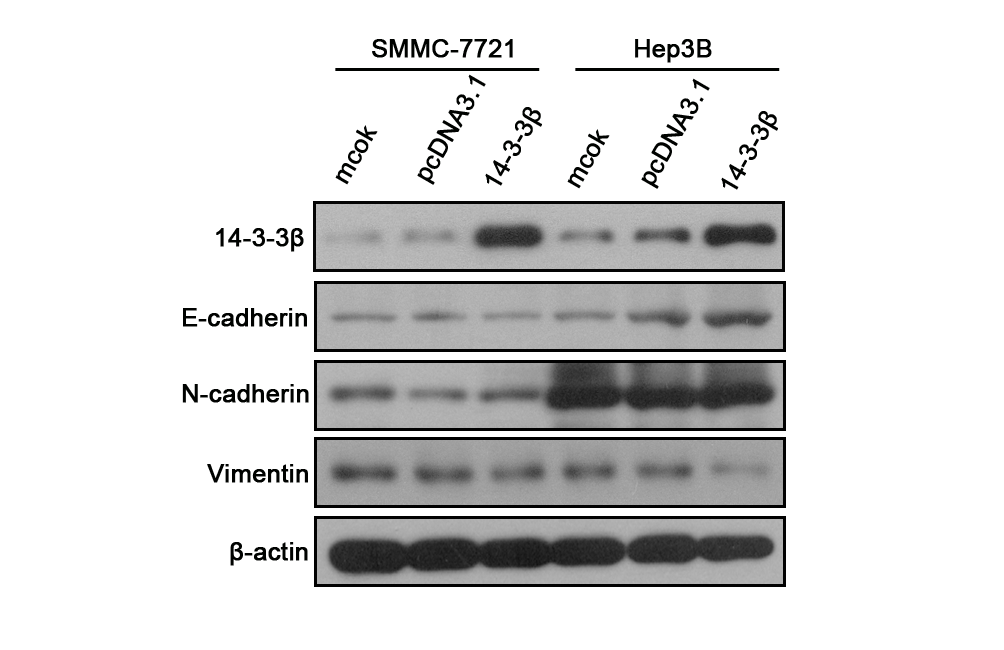

Supplement: S3 Fig — SMMC-7721 and Hep3B cells were transfected with 14-3-3β expression vector or pcDNA3.1 empty vector. The protein levels of E-cadherin, V-cadherin and Vimentin were analyzed by western blotting 24 h after transfection. (TIF) [file pone.0146070.s003.tif]

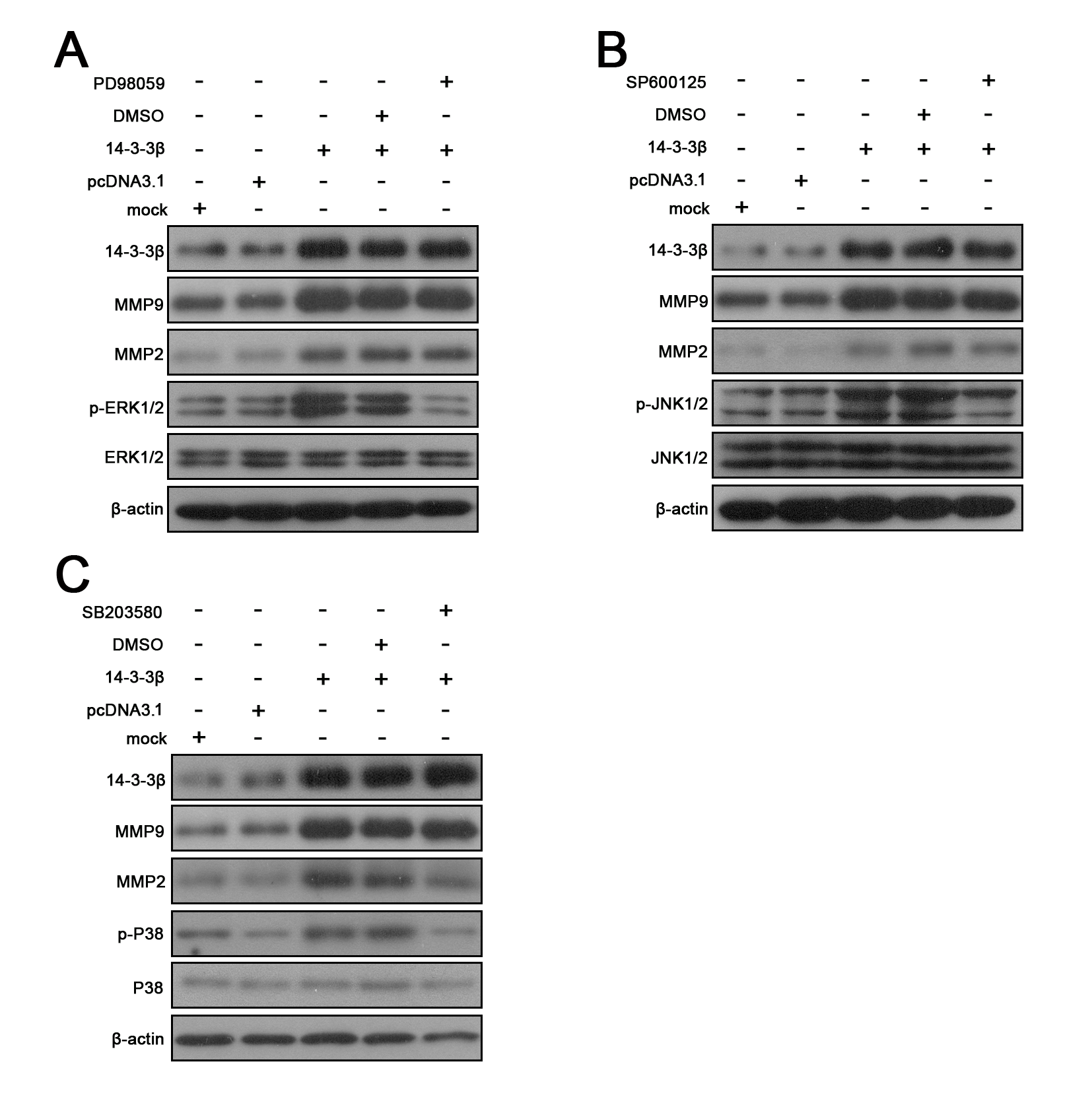

Supplement: S4 Fig — 14-3-3β- or pcDNA3.1-transfected Hep3B cells were treated with (A) 20 μM PD98059, a MEK inhibitor, (B) 20 μM SP600125, a JNK inhibitor, or (C) 10 μM SB203580, a P38/ERK2 inhibitor, for 5 h, and subjected to western blot analysis of the inhibitory effect on signal transduction and the expression of MMP2 and MMP9. (TIF) [file pone.0146070.s004.tif]

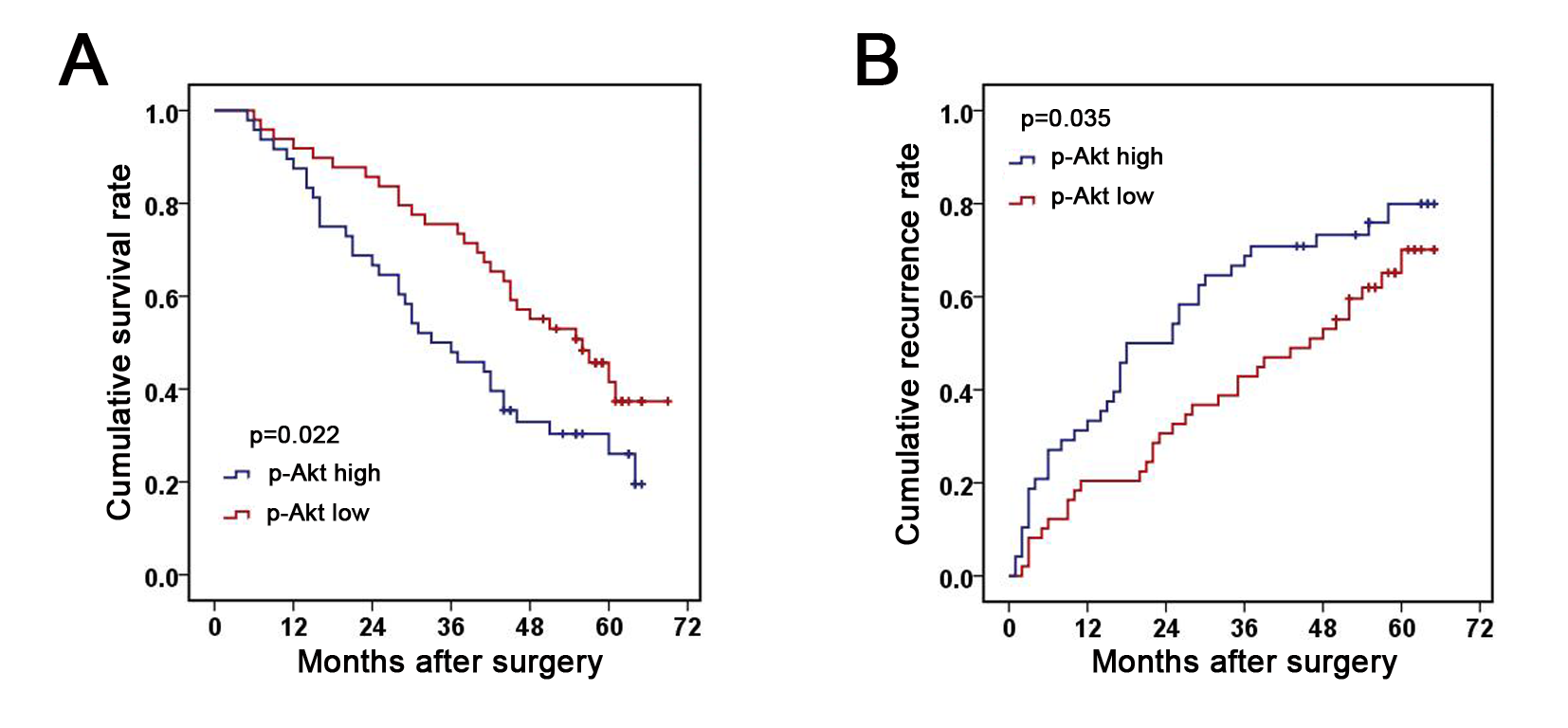

Supplement: S5 Fig — (A) Cumulative post-surgical survival rate and (B) cumulative post-surgical recurrence rate were analyzed by Kaplan-Meier curves in HCC patients with high or low levels of p-Akt. (TIF) [file pone.0146070.s005.tif]
