# Supplementary material for: 14-3-3β Promotes Migration and Invasion of Human Hepatocellular Carcinoma Cells by Modulating Expression of MMP2 and MMP9 through PI3K/Akt/NF-κB Pathway
Source: PLoS One. 2016 Jan 5;11(1):e0146070. doi: 10.1371/journal.pone.0146070 (PMC4711775; doi:10.1371/journal.pone.0146070)
Supplement: S1 Table — (DOCX) [file pone.0146070.s006.docx]

**S1 Table. Information about the antibodies used in Western blotting**

| Name | Supplier | Catalog number | Dilution factor | Polyclonal/ monoclonal | Host species |
| --- | --- | --- | --- | --- | --- |
| 14-3-3β | ABGENT | ALS13620 | 1:500 | Polyclonal | Rabbit |
| p-IκBα | Bioss | bs-18128R | 1:200 | Polyclonal | Rabbit |
| MMP2 | Wanleibio | WL1579 | 1:500 | Polyclonal | Rabbit |
| MMP9 | Wanleibio | WL02141 | 1:500 | Polyclonal | Rabbit |
| P38 | Wanleibio | WL00764 | 1:500 | Polyclonal | Rabbit |
| p-P38 | Wanleibio | WLP1576 | 1:500 | Polyclonal | Rabbit |
| JNK | Wanleibio | WL01295 | 1:500 | Polyclonal | Rabbit |
| p-JNK | Wanleibio | WL01813 | 1:500 | Polyclonal | Rabbit |
| ERK1/2 | Wanleibio | WL01864 | 1:1000 | Polyclonal | Rabbit |
| p-ERK1/2 | Wanleibio | WLP1512 | 1:1000 | Polyclonal | Rabbit |
| Akt | Wanleibio | WL01619 | 1:1000 | Polyclonal | Rabbit |
| p-Akt | Wanleibio | WLP001a | 1:500 | Polyclonal | Rabbit |
| NF-κB (p65) | Wanleibio | WL01980 | 1:1000 | Polyclonal | Rabbit |
| E-cadherin | Wanleibio | WL01482 | 1:500 | Polyclonal | Rabbit |
| N-cadherin | Wanleibio | WL01047 | 1:500 | Polyclonal | Rabbit |
| Vimentin | Wanleibio | WL01960 | 1:500 | Polyclonal | Rabbit |
| β-actin | Wanleibio | WL01774 | 1:1000 | Polyclonal | Rabbit |
| Lamin A | Santa Cruz | sc-71481 | 1:1000 | Monoclonal | Mouse |
| Anti-rabbit IgG | Beyotime | A0208 | 1:2000 | Polyclonal | Goat |
| Anti-mouse IgG | Beyotime | A0216 | 1:2000 | Polyclonal | Goat |
