# Supplementary material for: 14-3-3β Promotes Migration and Invasion of Human Hepatocellular Carcinoma Cells by Modulating Expression of MMP2 and MMP9 through PI3K/Akt/NF-κB Pathway
Source: PLoS One. 2016 Jan 5;11(1):e0146070. doi: 10.1371/journal.pone.0146070 (PMC4711775; doi:10.1371/journal.pone.0146070)
Supplement: S2 Table — (DOCX) [file pone.0146070.s007.docx]

**S2 Table. Clinicopathologic features of HCC patients**

| Features | Values/Counts |
| --- | --- |
| Sex (male/female) | 83/14 |
| Age (yrs), median (range) | 47(32-83) |
| HBVAg (positive/negative) | 97/0 |
| HBeAg (positive/negative) | 27/70 |
| Liver cirrhosis (yes/no) | (78/19) |
| AFP (ng/ml), median (range) | 69.9(1.3-1210) |
| ALT (U/L, mean±SD) | 76.66±200.57 |
| Tumor size (cm, mean±SD) | 5.14±3.65 |
| Tumor number (single/multiple) | 8/89 |
| Encapsulation (complete/no) | 23/74 |
| Microvascular invasion (yes/no) | 44/53 |
| PVTT (yes/no) | 9/88 |
| Tumor differentiation (II/III) | 29/68 |
| TNM stage (I/II/IIIa) | 68/13/18 |
| Early recurrence (yes/no) | 39/58 |
