# Supplementary material for: 14-3-3β Promotes Migration and Invasion of Human Hepatocellular Carcinoma Cells by Modulating Expression of MMP2 and MMP9 through PI3K/Akt/NF-κB Pathway
Source: PLoS One. 2016 Jan 5;11(1):e0146070. doi: 10.1371/journal.pone.0146070 (PMC4711775; doi:10.1371/journal.pone.0146070)
Supplement: S3 Table — (DOCX) [file pone.0146070.s008.docx]

**S3 Table. Density of intratumoral/peritumoral 14-3-3β staining**

|  | Density | | | *P* value |
| --- | --- | --- | --- | --- |
|  | Median | Q1-Q3 | Range |  |
| Intratumoral | 0.3571 | 0.3117-0.4005 | 0.1876-0.5237 | <0.001 |
| Peritumoral | 0.2689 | 0.2426-0.2937 | 0.1775-0.3854 |  |
