# Supplementary material for: 14-3-3β Promotes Migration and Invasion of Human Hepatocellular Carcinoma Cells by Modulating Expression of MMP2 and MMP9 through PI3K/Akt/NF-κB Pathway
Source: PLoS One. 2016 Jan 5;11(1):e0146070. doi: 10.1371/journal.pone.0146070 (PMC4711775; doi:10.1371/journal.pone.0146070)
Supplement: S4 Table — (DOCX) [file pone.0146070.s009.docx]

**S4 Table. Relationship between intratumoral 14-3-3β expression and survival time**

| Median time (months) | Relative 14-3-3β expression | | *P* value |
| --- | --- | --- | --- |
|  | Low | High |  |
| OS | 53 | 29.5 | <0.001 |
| TTR | 50 | 19.5 | <0.001 |
