# Supplementary material for: 14-3-3β Promotes Migration and Invasion of Human Hepatocellular Carcinoma Cells by Modulating Expression of MMP2 and MMP9 through PI3K/Akt/NF-κB Pathway
Source: PLoS One. 2016 Jan 5;11(1):e0146070. doi: 10.1371/journal.pone.0146070 (PMC4711775; doi:10.1371/journal.pone.0146070)
Supplement: S5 Table — (DOCX) [file pone.0146070.s010.docx]

**S5 Table. Relationship between intratumoral 14-3-3β expression and survival or recurrence rate**

| Rate |  | Relative 14-3-3β expression | | | *P* value |  |
| --- | --- | --- | --- | --- | --- | --- |
|  |  | Low | High | |  |  |
| 3-year OS (%) |  | 0.816±0.055 | | 0.417±0.071 | <0.001 |  |
| 5-year OS (%) |  | 0.518±0.074 | | 0.138±0.063 | <0.001 |  |
| 3-year TTR (%) |  | 0.388±0.070 | | 0.729±0.064 | <0.001 |  |
| 5-year TTR (%) |  | 0.571±0.080 | | 0.906±0.046 | <0.001 |  |
